# Clinical significance of FBXW7 loss of function in human cancers

**DOI:** 10.1186/s12943-022-01548-2

**Published:** 2022-03-26

**Authors:** Jingyi Fan, Marcia Bellon, Mingyi Ju, Lin Zhao, Minjie Wei, Liwu Fu, Christophe Nicot

**Affiliations:** 1 State Key Laboratory of Oncology in South China; Collaborative Innovation Center for Cancer Medicine, Guangdong Esophageal Cancer Institute; Sun Yat-sen University Cancer Center, Guangzhou, 510060 Guangdong Province China; 2 Department of Pharmacology, School of Pharmacy, China Medical University, Shenyang, 110122 China; 3 Liaoning Province, China Liaoning Key Laboratory of Molecular Targeted Anti-Tumor Drug Development and Evaluation, Liaoning Cancer Immune Peptide Drug Engineering Technology Research Center, Key Laboratory of Precision Diagnosis and Treatment of Gastrointestinal Tumors, Ministry of Education, China Medical University, Shenyang, 110122 Liaoning Province China; 4 Department of Pathology and Laboratory Medicine, Center for Viral Pathogenesis, University of Kansas Medical Center, 3901 Rainbow Boulevard, Kansas City, KS 66160 USA

**Keywords:** FBXW7, FBW7, CDC4, MYC, NOTCH, Cyclin E, MCL-1, Cancer, Tumor suppressor, Mutation, Epigenetic, Non-coding RNA, miRNA, circRNA, LncRNA, Apoptosis, DNA repair, Cell cycle, Chromosome instability, Centrosome, Aneuploidy, Immunotherapy, Drug resistance

## Abstract

FBXW7 (F-Box and WD Repeat Domain Containing 7) (also referred to as FBW7 or hCDC4) is a component of the Skp1-Cdc53 / Cullin-F-box-protein complex (SCF/β-TrCP). As a member of the F-box protein family, FBXW7 serves a role in phosphorylation-dependent ubiquitination and proteasome degradation of oncoproteins that play critical role(s) in oncogenesis. FBXW7 affects many regulatory functions involved in cell survival, cell proliferation, tumor invasion, DNA damage repair, genomic instability and telomere biology. This thorough review of current literature details how FBXW7 expression and functions are regulated through multiple mechanisms and how that ultimately drives tumorigenesis in a wide array of cell types. The clinical significance of FBXW7 is highlighted by the fact that FBXW7 is frequently inactivated in human lung, colon, and hematopoietic cancers. The loss of FBXW7 can serve as an independent prognostic marker and is significantly correlated with the resistance of tumor cells to chemotherapeutic agents and poorer disease outcomes. Recent evidence shows that genetic mutation of FBXW7 differentially affects the degradation of specific cellular targets resulting in a distinct and specific pattern of activation/inactivation of cell signaling pathways. The clinical significance of FBXW7 mutations in the context of tumor development, progression, and resistance to therapies as well as opportunities for targeted therapies is discussed.

## Background

The FBXW7 gene is located at chromosome 4q31q.3 and consists of 4 untranslated introns and 13 coding exons. There are three isoforms of FBXW7, namely FBXW7-α, FBXW7-β and FBXW7-γ that are predominantly localized in the nucleus, cytoplasm, and nucleolus, respectively. Their structural divergence is manifested by differences in the first exon, while the role of these isoforms has been previously reviewed [1]. The alpha form is the most abundant isoform and accounts for the most substrate turnover. Structurally FBXW7 is composed of seven WD40 domains involved in substrate binding, and a dimerization domain and F-box domain involved in the recruitment of SCF components for E3 ligase activity. FBXW7 interacts with substrates through a conserved sequence known as the CDC4 phosphodegron (CPD) motif. Glycogen synthase kinase 3 beta (GSK3β)-mediated phosphorylation of cellular targets is a required step for the recruitment of FBXW7, ubiquitination, and proteasome degradation of substrates [2]. FBXW7 can function as monomer or as a dimer and some studies have suggested that this may affect target selection and binding strength. In addition, dimerization of FBXW7 may play important roles in cancer cells whereby one copy is mutated. In fact, several studies have shown dominant negative functions for mutated FBXW7 alleles. FBXW7 is frequently inactivated in human cancers through genetic and epigenetic mechanisms, along with post transcriptional modifications. Subsequently, inactivation of FBXW7 is a major cause of carcinogenesis and the development of human cancers. Numerous studies have demonstrated that loss of FBXW7 is strongly associated with carcinogenesis, tumor metastasis, poorer outcomes in cancer patients, and resistance to chemo-, radiation-, and immuno-therapies. Therefore, FBXW7 expression is an independent prognostic biomarker of poorer outcomes in some cancers.

This review will focus on current and past literature. The regulation of FBXW7 through transcriptional and post-transcriptional means will be highlighted first, to get a better appreciation of the complex regulatory role of FBXW7. Known FBXW7 target proteins will then be discussed, highlighting their correlation with known oncogenic pathways involved in carcinogenesis. Finally, a detailed analysis of the current understanding of FBXW7 in individual cancers, including current therapies used to target FBXW7 will de deliberated.

### The regulation of FBXW7 expression in normal and cancer cells

#### Transcriptional regulation of FBXW7

**Fig. 1:**
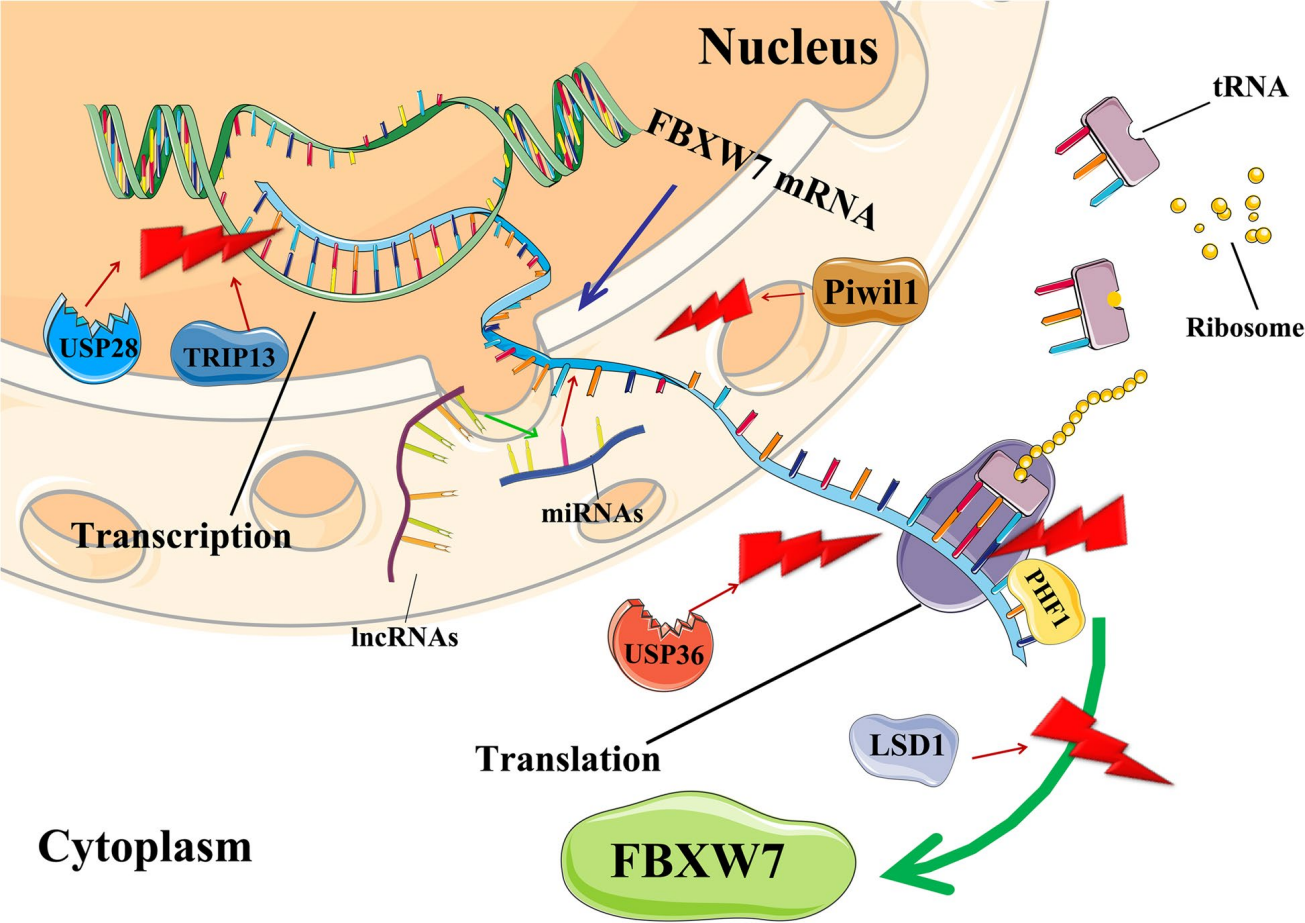
FBXW7 Regulation in the Cell. Multiple FBXW7-related expression regulators regulate FBXW7 expression in different biological processes. lncRNAs can act as sponges that bind to miRNAs and restrain the effect of miRNAs on FBXW7 expression. When the mRNA of FBXW7 is looped by the action of other loop RNAs or looped by itself, the above-mentioned FBXW7 expression regulators no longer have an effect on the expression of FBXW7

Studies have shown that multiple transcription factors can regulate the expression of FBXW7 isoforms [3–9]. There is a diverse array of FBXW7 transcriptional regulators that include known transcriptional activators (TP53 and C/EBPδ), transcriptional repressors (PHF1 and Hes5), ligands (TGFβ) and ATPase super family (TRIP13) proteins. Most of these factors negatively regulate FBXW7 expression. TRIP13 (Thyroid Hormone Receptor Interacting Protein 13) belongs to a protein superfamily that is overexpressed in many human cancers and is associated with poor prognosis in cancer patients [10]. Among the many roles of TRIP13, are involvement in the DNA damage response and the spindle assembly checkpoint (SAC) [11]. TRIP13 can also bind directly to the FBXW7 promoter and can mediate transcriptional repression of the FBXW7 promoter (Fig. 1) [10, 12]. An additional factor that directly interacts with the FBXW7 promoter region is PHF1 (PHD finger protein 1) (Fig. 1). PHF1 is a reader for histone arginine methylation, which targets PHF1 to the chromatin. PHF1 binds to the promoter region of FBXW7 and represses transcription via a complex that consists of PHF1, a methyltransferase (PRMT5; protein arginine methyltransferase 5), and ubiquitin ligase (Cullin 4B) complex [13]. Like PHF1, Hes5 (hes family bHLH transcription factor 5) is also a known transcriptional repressor. Hes5 acts downstream of NOTCH signaling and suppresses FBXW7 expression resulting in a negative regulatory loop [14]. In addition, Hes5-mediated suppression of FBXW7 has also been linked to TGIF1 (Transforming Growth Factor-Beta-Induced Factor) degradation and inactivation of the TGF-β signaling pathway [15].

Several known transcriptional activators also play roles in FBXW7 transcriptional regulation. Though C/EBPδ (CCAAT/enhancer-binding protein-δ) usually activates transcription, a reverse role has been shown for FBXW7 transcription. C/EBPδ directly inhibits the FBXW7 promoter and enhances the mTOR/AKT/S6K1 signaling pathway. This leads to augmented translation and activity of HIF-1α (hypoxia-inducible factor-1α), with a resulting increase in cell migration and metastasis [3]. In contrast to C/EBPδ, C/EBPα and C/EBPβ appear to have no effect on FBXW7 promoter expression. Several studies have also reported a regulatory loop between the tumor suppressor p53 (TP53) and FBXW7 [16, 17]. The first exon of FBXW7 contains a p53-binding site which acts as a positive regulator of transcription [7]. However, in response to DNA damage induced activation of ATM (ATM Serine/Threonine Kinase), p53 is phosphorylated. This action generates a signal for FBXW7 interaction and degradation thereby creating a negative feedback regulatory loop [17]. Under normal physiological conditions p53-mediated activation of FBXW7 plays essential roles in the control of cell cycle and DNA strand break repair.

#### Epigenetic regulation of FBXW7

**Fig. 2:**
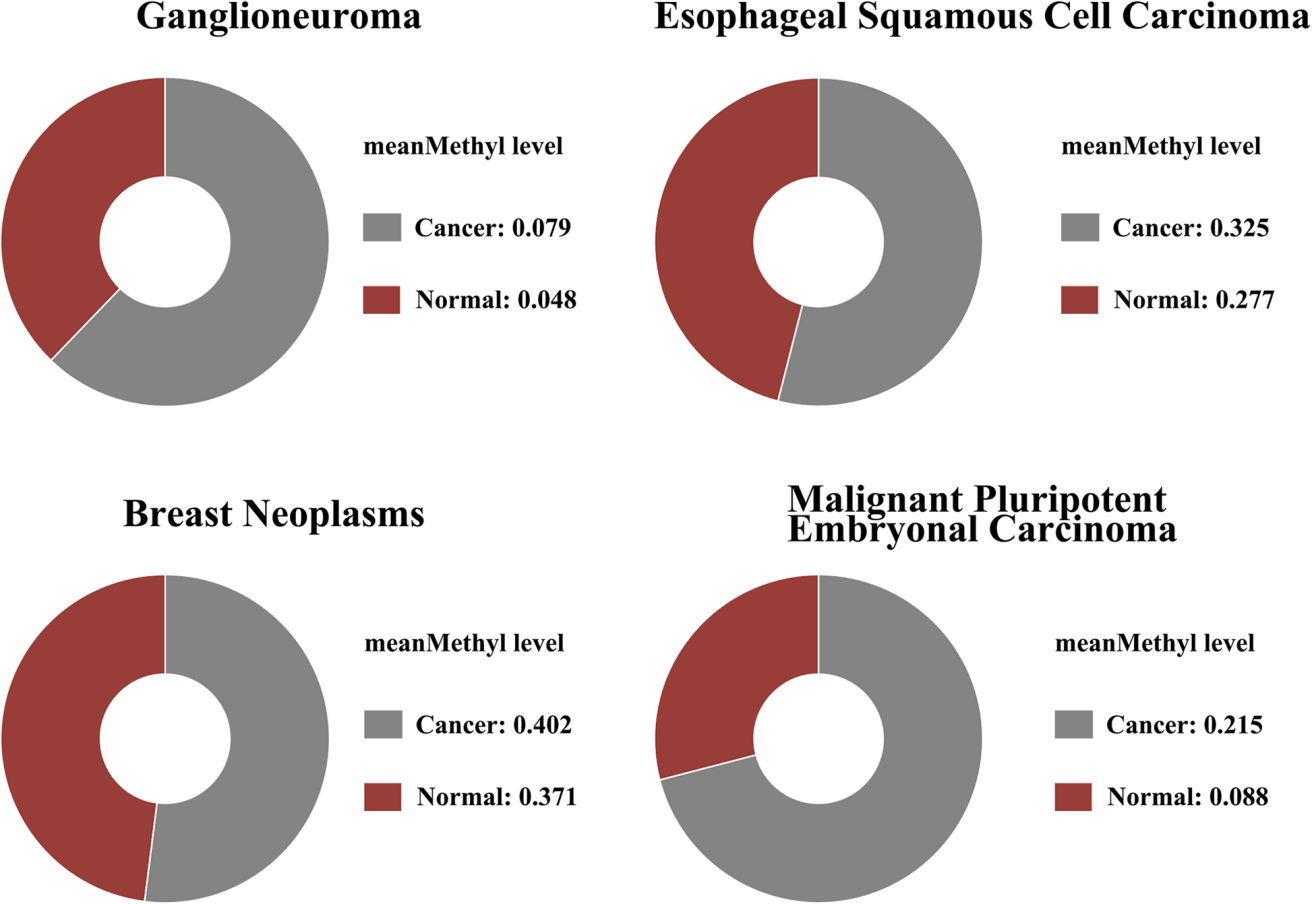
Methylation Profile of FBXW7. Representation of the methylation profile of FBXW7 in different cancers and how it affects disease progression (http://gdata.hrbmu.edu.cn/diseasemeth/index.html)

Epigenetic inactivation of tumor suppressor genes by promoter hypermethylation is a frequent event during tumorigenesis [18]. The Human Disease Methylation Database demonstrates an inferred or potential disease association between FBXW7 methylation and several diseases (Fig. 2). This is especially true for malignant pluripotent embryonal carcinoma. Among the three isoforms of FBXW7, epigenetic regulation of FBXW7-β through methylation is the most common. This is likely due to the differential expression of FBXW7-β in tissues and cells. The promoter of FBXW7-β has been reported to be epigenetically regulated by DNA and histone modifications and is methylated in a variety of cancers, particularly in primary breast cancer cells [19]. Further studies demonstrate a role for FBXW7 methylation in thymic tumors. Methylation levels of FBXW7-β differ among progressive tumor samples; and methylation of FBXW7-β is positively correlated with tumor malignancy. This suggests that methylation inhibition of FBXW7-β is a potential cause of thymic tumor progression [20–22].

As discussed above, PRMT5 is a methyltransferase involved in PHF1 regulation of FBXW7 transcriptional levels. PRMT5 symmetrically links two methyl groups onto arginine residues on histones H3 and H4. These marks are generally associated with transcriptional repression [23]. Studies have shown that PRMT5 increases the occupancy of heterochromatin markers H4R3me2 and H3K9me3 and decreases the occupancy of the euchromatin marker H3K9ac onto the FBXW7 promoter. This results in epigenetic silencing of FBXW7 mRNA expression [24]. In fact, PRMT5 expression is inversely correlated with FBXW7 and closely associated with aberrant MYC function in human cancers [25]. This suggests that PRMT5, or related epigenetic factors, may represent potential therapeutic targets in a subset of tumors bearing silenced wild type FBXW7 gene.

#### FBXW7 regulation at the mRNA level

RNA stability, mRNA degradation, and translation can be regulated by the m^6^A methyltransferase complex. N6-methyladenosine (m^6^A) is a reversible, ubiquitous mRNA methylation modification. This m^6^A methyltransferase complex contains several enzymes that are known as “writers”, adding m^6^A onto mRNA [26]. One of these enzymes, METTL3 (Methyltransferase-Like Protein 3), can stimulates m^6^A methylation in the mRNA coding sequence of FBXW7 [27]. This leads to increased translational efficiency of FBXW7 mRNA, allowing METTL3 to act as a positive regulator of FBXW7.

Piwi is a subfamily of Argonaute proteins with important roles in stem cells, differentiation, and epigenetic regulation. Piwi proteins can bind small RNAs. Among the Piwi family, PiwiL1 (Piwi Like RNA-Mediated Gene Silencing 1), is overexpressed in glioma stem-like cells (GSCs). In GSCs, PiwiL1 was found to negatively regulate the mRNA stability of FBXW7 [28] (Fig. 1). Unlike its role with other mRNAs, PiwiL1 did not bind to FBXW7 mRNA directly, and was likely inhibiting FBXW7 indirectly through another mRNA.

#### Non-coding RNA Regulation of FBXW7

Multiple non-coding RNAs (ncRNAs) also regulate the expression of FBXW7 [29]. While some ncRNAs are essential for standard protein translation, including transfer RNAs (tRNAs) and ribosomal RNAs (rRNA), other ncRNAs have gene regulatory roles, include microRNAs (miRNAs), long non-coding RNAs (lncRNAs), and circular RNAs (circRNAs). In general, ncRNAs are not translated into protein; however, some circRNAs have been shown to encode smaller forms of the parent protein. MicroRNAs play an important regulatory role in tumorigenesis and development [30–32]. By interacting with the 3'UTR of target mRNAs, miRNAs achieve post-transcriptional repression of mRNA translation, thereby regulating many biological processes including proliferation, apoptosis, invasion/metastasis, and angiogenesis [30]. miRNAs act mostly as negative regulators of FBXW7 expression and generally display a pro-oncogenic role. Numerous miRNAs have been shown to target FBXW7 in various cells, tissues, and cancers. Among these are miR-25-3p, miR-32, miR-92b, miR-96, miR-155-3p, miR-182, miR-223, and miR-367. An expanded list of miRNAs targeting FBXW7 is included in Table 1. It is likely this list will grow as the study of FBXW7-miRNA interaction(s) expands. While each of these FBXW7-targeting miRNAs were discovered in individual cancers, it is still unknown whether they possess broad regulatory roles for FBXW7 expression in all cancers. Several studies have demonstrated a roll for miR-223 in regulating FBXW7 expression, suggesting miR-223 has a broad capacity to target FBXW7 across various tissues and disease. TAL1 (T-Cell Acute Lymphocytic Leukemia Protein 1), one of the most frequently dysregulated oncogenes in T-ALL (T-cell acute lymhoblastic leukemia), has been shown to induce expression of miR-223 and repress expression of FBXW7 [33]. In addition, miRNA-223-induced inhibition of the FBXW7 has been shown in solid tumors as well. miR-223 affects the proliferation and apoptosis of colorectal cancer cells via activation of the NOTCH and AKT/mTOR pathways [33–36]; and inhibits FBXW7 in oesophageal squamous cell carcinoma [37]. MYC-regulated miRNA-92b and miR-92a have also been shown to inhibit FBXW7 expression in colorectal cancer cells [38, 39]. The same is true for miR-92a regulation of FBXW7 in hepatocellular carcinoma (HCC); where overexpression of miR-25-3p and miR155-3p have also been shown to suppress FBXW7 mRNA expression, promoting the migration and invasion of tumor cells [40].

**Table 1:** Non-coding RNA regulation of FBXW7

RNA type	ncRNA	Research subject	FBXW7 expression	Results or effects of the research	References
miRNAs	miR-18b-3p	Hepatic fibrosis	down-regulated	Promote liver fibrosis, promote tumor proliferation, and promote inflammation	[41]
	miR-23a	Colorectal cancer	down-regulated	Promote malignant transformation of colorectal cancer	[42]
	miR-25	Hepatocellular Carcinoma	down-regulated	Invasion and migration	[43]
	miR-25	Prostatic Small Cell Neuroendocrine Carcinoma	down-regulated	Upregulate AURKA, promote tumor proliferation	[44]
	miR-27a	Bronchial epithelial cell carcinoma	down-regulated	Malignant transformation induced by the viral oncoprotein ST	[45]
	miR-27b-3p	Multiple myeloma	down-regulated	Mcl-1 is upregulated and produces apoptosis resistance	[46]
	miR-32	Breast cancer	down-regulated	Inhibits apoptosis and promotes proliferation and migration of breast cancer cells.	[47]
	miR-92a	Cervical Cancer	down-regulated	Promote proliferation of cervical cancer cells	[48]
	miR-92a-3p	Colorectal cancer	down-regulated	Activation of Wnt/β-catenin pathway promotes cell stemness, EMT, metastasis and 5-FU/L-OHP resistance in CRC	[39]
	miR-92a-3p	Gastric cancer	down-regulated	Promote invasive migration of gastric cancer	[49]
	miR-92a-3p	Hepatocellular carcinoma	down-regulated	Promotes proliferation and cell cycle transition from G1 to S phase, and inhibits apoptosis in HCC cells	[40]
	miR-101	Bone marrow mesenchymal stem cell	down-regulated	Upregulate HIF1α	[50]
	miR-144	Nasopharyngeal carcinoma	down-regulated	Upregulate HIF1α	[51]
	miR-155	Glioma	down-regulated	Enhance the tumor cell viability	[52]
	miR-155-3p	Hepatocellular carcinoma	down-regulated	Promote HCC cell proliferation	[53]
	miR-182	Breast cancer	down-regulated	Upregulate the HIF-1α-VEGF-A axis and promotes tumor invasion and migration	[54]
	miR-182	Colon adenoma	down-regulated	Induced transformation of adenoma to adenocarcinoma	[55]
	miR-197-3p	Gastric cancer	down-regulated	Up-regulate cyclinD1, EMT, YAP1, down-regulate CCDC6	[56]
	miR-223	Gastric cancer	down-regulated	Promote the cisplatin resistance; reduces trastuzumab sensitivity	[36, 57]
	miR-223	Oesophageal squamous cell carcinoma	down-regulated	Intervention of poor patient prognosis	[37]
	miR-223	Colorectal cancer	down-regulated	Increases endogenous cell cycle protein E protein and activity	[58]
	miR-367	Lung cancer	down-regulated	Promotes tumor cell invasion and migration and is associated with poor prognosis	[59]
	miR-367	Hepatocellular carcinoma	down-regulated	Promote EMT	[60]
	miR-500a-3p	Gastric cancer	down-regulated	Increase cisplatin resistance	[61]
	miR-503	Colon adenoma	down-regulated	Induced transformation of adenoma to adenocarcinoma	[55]
	miR-586	Lung cancer	down-regulated	Promote tumorigenesis	[62]
	miR-5000-3p	Laryngocarcinoma	down-regulated	Promote the invasion and migration of laryngeal cancer cells	[63]
lncRNAs	lncRNA-CASC2	Hepatocellular carcinoma	up-regulated	Inhibit EMT	[60]
	lncRNA-MALAT1	Glioma	up-regulated	Suppresse tumor cell viability	[52]
	lncRNA-MIF	Lung cancer	up-regulated	Inhibit cMyc, inhibits aerobic glycolysis and tumorigenesis	[62]
	lncRNA-MIR22HG	Laryngocarcinoma	up-regulated	Inhibit the invasion and migration of laryngeal cancer cells	[63]
	lncRNA-MT1JP	Gastric cancer	up-regulated	Inhibit invasive migration of gastric cancer	[49]
	lncRNA-FRE1L4	Gastric cancer	up-regulated	Inhibit prostate cancer progression	[49]
	lncRNA-TTN-AS1	Esophageal Squamous Cell Carcinoma	up-regulated	Competitive binding of miR-133b promotes the expression of transcription factor Snail1, leading to an EMT cascade response	[64]
circRNAs	hsa_circ_001988^a^	Gastric cancer	up-regulated	Up-regulate cyclinD1, EMT, YAP1, down-regulate CCDC6	[56]
	circFBXW4	Hepatic fibrosis	up-regulated	Inhibit liver fibrosis, inhibit tumor proliferation, anti-inflammatory	[41]
	FBXW7-185aa	Glioma	-	Reduce the expression of c-Myc expression, associated with good patient survival	[65]
	circ-FBXW7^a^	Colon adenoma	-	Decrease the expression of NEK2, mTOR and PTEN to induce tumor suppression	[66]

The table describes all currently known regulatory roles of non-coding RNA (ncRNA), including miRNAs, lnc-RNAs and circ-RNAs, affecting FBXW7 expression which can be up-regulated or down-regulated. The cancer type and published biological consequences of FBXW7 alterations is indicated in the second and fourth columns. References for which the ncRNA was described is listed on the right end-side of the table. ^a^For cirRNAs, circ-FBXW7 goes by several names, including has_circ_001988 and circ_022705

SREBP2 is a member of the Sterol Regulatory Element-Binding Protein (SREBP) family, which regulates cell survival and lipid synthesis. SREBP2 induces miR-96 and miR-182 that directly suppress its negative regulator FBXW7, which results in a positive feed forward loop [67]. miR-182 has also been associated with the progression of glioma and breast cancers by directly targeting FBXW7 [68, 69]; again highlighting the broad role indiviudal miRNAs play in regulating FBXW7 across various cancers. High levels of miR-32 expression downregulate FBXW7, promote cell proliferation, migration and suppress apoptosis in breast cancer [47]. Finally, miR-367, an indicator of a poorer prognosis in NSCLC patients, stimulates tumor growth through direct inhibition of FBXW7 [59]. miR-367 also inhibits FBXW7 in HCC.

Long non-coding RNAs work intimately with miRNAs in regulating protein expression. lncRNAs can regulate the function of miRNAs by acting as a competing endogenous RNA (ceRNA) [70]. Consequently, any lncRNA that regulates the aforementioned miRNAs would be expected to act as positive regulators of FBXW7 expression and reduce the expression of FBXW7 targets. ceRNA activity can be affected by tissue expression restrictions, lncRNA abundance, subcellular localization of ceRNA components, the binding affinity of miRNAs to their targets, and through RNA modifications [71]. While expression of lncRNAs may have tumor suppressor effects, a consideration of the complexity of intertwined positive and negative regulators of different signaling pathways and in distinct tissues should be considered. lncRNA TTN-AS1 acts as sponge for miR-15b-5p to upregulate FBXW7 expression and to inhibit the proliferation and promote apoptosis of ovarian cancer cells [72]. This represents an alternative suppressor function of TTN-AS1, which is generally viewed as a pro-oncogenic lncRNA in many human cancers [64]. Similarly, while lncMALAT-1 overexpression can have tumor-suppressing effects in glioma cells through ceRNA of miR-155 and increased expression of FBXW7, lncRNA MALAT1 contributes to non-small cell lung cancer development via modulation of the miR-124/STAT3 axis [73]. lncRNA-MIF is a c-MYC-activated lncRNA which targets miR-586 and increases FBXW7 expression creating a feedback regulatory loop for c-MYC expression [62]. Sponging of miR-92a-3p by lncRNA FER1L4 and lncRNA MT1JP has also been shown to increase expression levels of FBXW7 [49, 74]. The FER1L4/miR-92a-3p/FBXW7 axis controls apoptosis signaling in prostate cancer [74]. Although expression of miR-92a-3p is elevated in gastric cancers, the ceRNA activity of lncRNA MT1JP can efficiently prevent miR-92a-3p repression of FBXW7 mRNA expression and induce apoptosis of gastric cancer cells [75]. MT1JP is down-regulated in gastric cancer tissues and may serve as a disease progression biomarker and a potential therapeutic strategy [49].

Circular RNAs are single stranded RNAs, and unlike other lncRNAs, form a stable, covalently closed circle [76]. circRNAs have multiple functions that include protein coding and gene regulation. One of the better studied circRNAs that regulates FBXW7 is circ-FBXW7 (also known as hsa_circ_001988 and has_circ_022705). Circ-FBXW7 is down-regulated in primary patient cells from glioblastoma, colorectal, and gastric cancer samples, and in triple-negative breast cancer cells [56, 65, 66, 77]. A similar lncRNA, circFBXW4, also targets FBXW7 and is down-regulated in hepatic fibrosis [41]. One of the main actions of circRNAs is to sponge away miRNAs. Circ-FBXW7 works by sponging miR-18b-5p and miR-197-3p; and circFBXW4 sponges miR-18b-3p in various cancers and disease processes [41, 56, 77, 78]. All these regulatory actions lead to down-regulation of FBXW7. Among the down-stream effects of increased circ-FBXW7 are decreased proliferation and increased apoptosis and chemoresistance [78]. Interestingly, circ-FBXW7 also encodes a novel protein, FBXW7-185 aa [65]. This 22kDa protein was positively associated with glioblastoma patient overall survival and inhibited proliferation with cell cycle arrest in glioma cells [65].

#### Genetic alterations of FBXW7

**Fig. 3:**
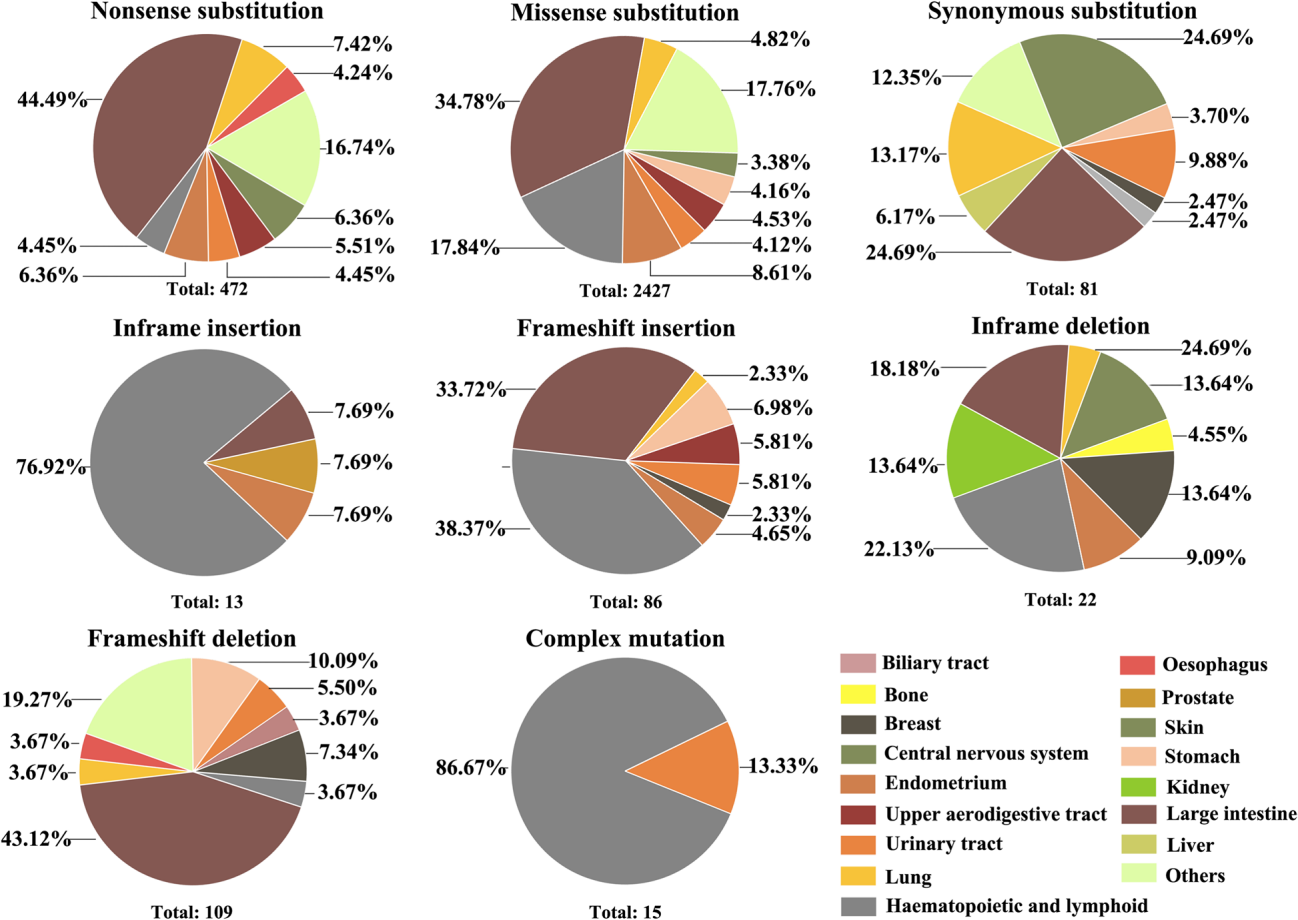
FBXW7 Genetic Mutations in Various Cancer Types. The FBXW7 gene occurs in a variety of mutation patterns including nonsense mutations, deletion mutations, and mixed mutation patterns that show different frequencies in different tumor types. Here is the distribution of different mutation patterns of FBXW7 in tissues

Inactivation of FBXW7 by genetic mutation, genomic deletion, or promoter hypermethylation is a major cause of carcinogenesis and development of human cancers. FBXW7 demonstrates a high mutation rate in many cancers [1]. Among all family members containing F-box and WD repetitive structural domains, FBXW7 exhibits the highest mutational frequency. Meta-analysis of the COSMIC database shows that the overall mutational rate of FBXW7 in all human tumors is 7.79% (number of mutated samples n=3844 / total number of samples n=49348). Among all mutation cases in malignant tumors, 72.70% of FBXW7 mutations are missense substitutions, 13.82% are nonsense substitutions, and 7.89% are insertion/deletion mutations [79]. Figure 3 represents the percentage of occurrence of different FBXW7 alterations in various human cancer types. Almost 50% of missense and nonsense mutations are observed in hematopoietic and large intestine related cancers (Fig. 3). Although the reported numbers are relatively small, is it intriguing that the distribution of mutations in large intestinal cancer is similar between frameshift insertions and frameshift deletions while hematopoietic cancers display a 10-fold higher frequency in frameshift insertions versus frameshift deletions (Fig. 3). The FBXW7 mutations mentioned above most often occur in the FBXW7 WD40 domain [80]. Three hotspot, pathogenic, mutations have been identified in residues Arg 465, Arg 479, and Arg 505 (also known as FBXW7AGE mutations). These conserved arginine residues are present in the WD40 structural domain and may be important for proper folding of the substrate binding pocket. However, while these mutants were initially thought to be “dead” mutants, unable to interact with any targets, recent studies demonstrate that these mutants have distinct phenotypes. Among them, R465C is the most frequently detected FBXW7 mutation in human primary tumors [81].

**Fig. 4:**
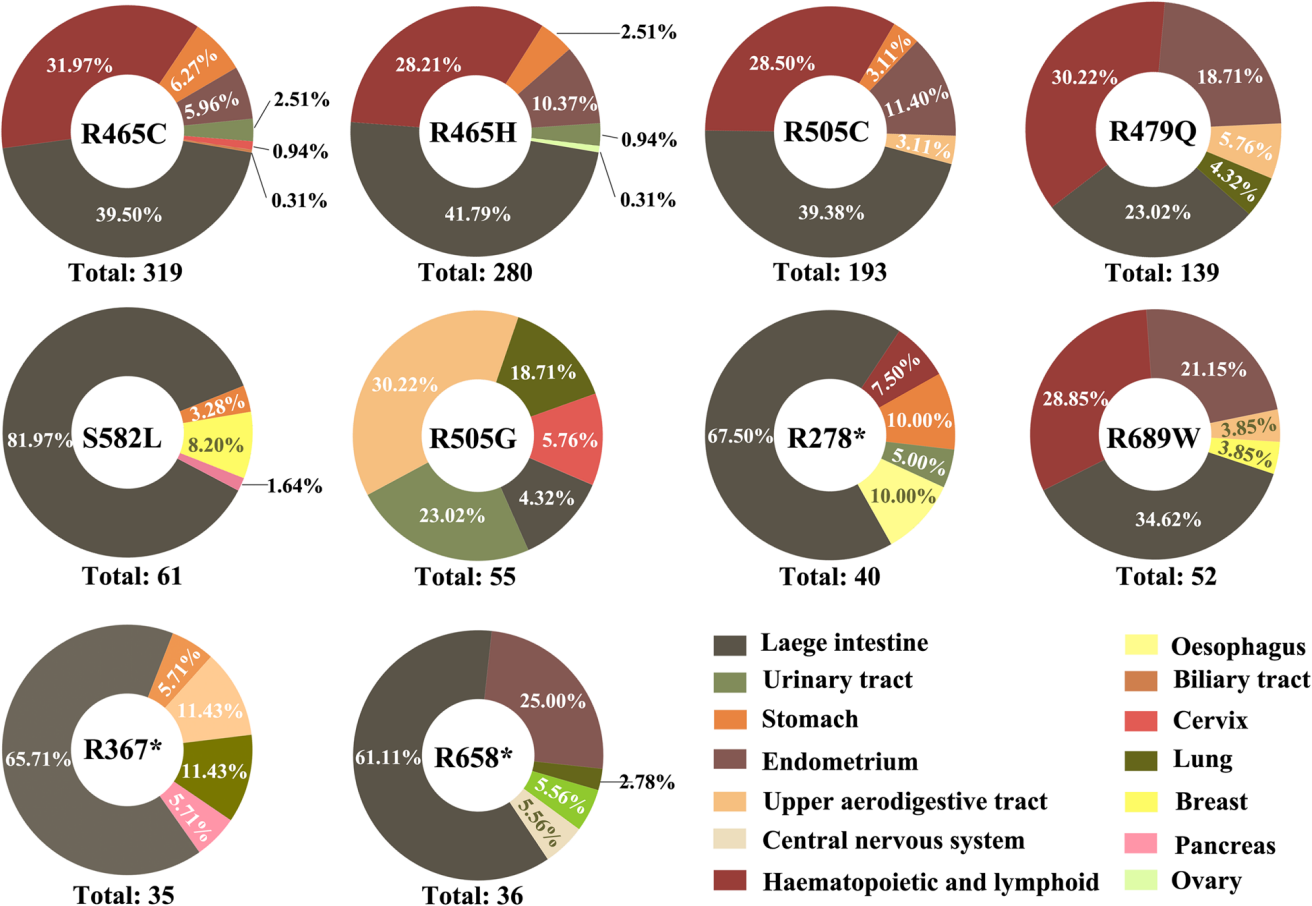
Distribution of FBXW7 Genetic Mutations in Cancer Types. Pie chart graphical distribution showing the relative percentage of different FBXW7 mutants most frequently found in human cancers

To better demonstrate the disease distribution and probability of occurrence of FBXW7 mutations, we performed statistical analysis using the cBioportal database [82, 83] (Table 2). Even though the rate of some FBXW7 mutations is relatively low in some cancer types, the fact that identical mutations are found across multiple cancer types suggests that these are relevant and warrant additional studies. For instance, L443H was found in hematopoietic and cervical cancers [84], while H460R was detected in both hematopoietic and gastric cancers [85]. According to our analyses from the COSMIC database data, the distribution of the most frequently found FBXW7 mutations in cancers is intriguingly different in different tissues (Fig. 4). The significance and role of each of these mutations in human cancer has not been investigated in depth and warrants future studies. While hot spot mutations, R465C, R465H, R505C, and R479Q, share similar distribution patterns, it is noticeable that R505G has a significantly different distribution. R505G is mostly detected in upper aerodigestive tract cancers (30.22%), urinary tract cancers (23.02%) and lung cancers (18.7%). Aerodigestive tract cancers were 10 times less frequent for R505C mutation (3.11%). Lung cancer and urinary tract cancers rarely had the FBXW7 mutation, R505C, with less than 1% of all cancers having this mutation. The fact that R505C and R505G have different representation among human cancers is likely a reflection of the differences in the ability of these mutants to bind and stimulate target degradation. Although the numbers are small, it is intriguing that some mutations appear to be more frequent in specific cancers. For example, cervical cancers were associated with R505G or R658*, breast cancer with S582L, oesophageal cancers with R278*, pancreatic cancer with R367*, and glioblastoma with R658* mutations. Additional studies that include larger cohorts are needed to validate these observations. In contrast, large intestinal cancers are equally distributed across FBXW7 mutations, except for R505G.

**Table 2:** Most frequent FBXW7 mutations in human tumors

Protein Change	Cancer Type	Mutation Type	Mutation Frequency	Sample Size
R14Q	Colon Adenocarcinoma	Missense	0.34	5120
X195_splice	Uterine Endometrioid Carcinoma	Splice	0.42	1078
	Stomach Adenocarcinoma	Splice	0.31	1351
R224Q	Uterine Endometrioid Carcinoma	Missense	0.3	8236
G423V	Uterine Serous Carcinoma/Uterine Papillary Serous Carcinoma	Missense	0.3	185
R441Q	Uterine Endometrioid Carcinoma	Missense	0.3	13882
R465C	Rectal Adenocarcinoma	Missense	0.43	1081
	Uterine Endometrioid Carcinoma	Missense	0.42	7291
	Stomach Adenocarcinoma	Missense	0.33	638
	Colon Adenocarcinoma	Missense	0.31	1119
R465H	Mucinous Adenocarcinoma of the Colon and Rectum	Missense	0.61	1622
	Uterine Endometrioid Carcinoma	Missense	0.44	1558
	Stomach Adenocarcinoma	Missense	0.43	2689
	Serous Ovarian Cancer	Missense	0.41	799
	Rectal Adenocarcinoma	Missense	0.39	9464
	Colon Adenocarcinoma	Missense	0.37	1208
R479G	Cervical Squamous Cell Carcinoma	Missense	0.6	167
	Bladder Urothelial Carcinoma	Missense	0.41	165
R479L	Head and Neck Squamous Cell Carcinoma	Missense	0.62	257
R479P	Cervical Squamous Cell Carcinoma	Missense	0.33	162
R479Q	Uterine Endometrioid Carcinoma	Missense	0.6	1322
	Uterine Mixed Endometrial Carcinoma	Missense	0.45	816
R505C	Cervical Squamous Cell Carcinoma	Missense	0.53	296
	Uterine Endometrioid Carcinoma	Missense	0.48	4835
	Stomach Adenocarcinoma	Missense	0.43	1705
	Uterine Serous Carcinoma/Uterine Papillary Serous Carcinoma	Missense	0.38	1512
	Esophageal Adenocarcinoma	Missense	0.38	1709
R505G	Endocervical Adenocarcinoma	Missense	0.75	1112
	Bladder Urothelial Carcinoma	Missense	0.61	416
	Colon Adenocarcinoma	Missense	0.5	117
	Lung Squamous Cell Carcinoma	Missense	0.33	335
	Cervical Squamous Cell Carcinoma	Missense	0.33	672
R505H	Uterine Endometrioid Carcinoma	Missense	0.54	450
	Colon Adenocarcinoma	Missense	0.34	108
R505L	Head and Neck Squamous Cell Carcinoma	Missense	0.56	345
D520E	Uterine Endometrioid Carcinoma	Missense	0.41	7858
D520N	Bladder Urothelial Carcinoma	Missense	0.38	530
D520Y	Colon Adenocarcinoma	Missense	0.27	5120
Y545C	Breast Invasive Ductal Carcinoma	Missense	0.72	171
	Head and Neck Squamous Cell Carcinoma	Missense	0.43	79
	Uterine Endometrioid Carcinoma	Missense	0.4	63
	Uterine Endometrioid Carcinoma	Missense	0.36	498
S546L	Bladder Urothelial Carcinoma	Missense	0.76	1294
S582L	Rectal Adenocarcinoma	Missense	0.7	138
	Colon Adenocarcinoma	Missense	0.4	169
R658*	Mucinous Adenocarcinoma of the Colon and Rectum	Nonsense	0.7	712
R658*	Rectal Adenocarcinoma	Nonsense	0.41	11437
	Uterine Endometrioid Carcinoma	Nonsense	0.37	6856
	Uterine Carcinosarcoma/Uterine Malignant Mixed Mullerian Tumor	Nonsense	0.21	10826
R658Q	Uterine Endometrioid Carcinoma	Missense	0.47	7801
	Colon Adenocarcinoma	Missense	0.41	2137
	Cervical Squamous Cell Carcinoma	Missense	0.41	1301
R689W	Colon Adenocarcinoma	Missense	0.37	176
	Uterine Endometrioid Carcinoma	Missense	0.36	12787

Based on the analysis of the TCGA database data displayed by cBioportal, we have categorized and summarized the most frequent mutations of FBXW7 and the cancer types in which the different types of mutations are most prevalent. The probability of mutation occurrence in each individual type of cancer (http://www.cbioportal.org/) is listed. The table indicates the nature of the mutation (missense, nonsense or splice defect). The sample size represents the number of tumors analyzed in search of a particular mutation.

Current studies demonstrate that some mutations in FBXW7 do not exhibit a complete loss of function and demonstrate specific selectivity for ubiquitinated substrates. Among them, the S462P, H468R, R465C and R479Q mutations of FBXW7 show similar effects and fail to induce degradation of any known target. In contrast, R505C retains the ability to bind to and degrade BRAF (B-Raf Proto-Oncogene, Serine/Threonine Kinase), but not NOTCH1, c-MYC, cyclin E, or MCL1. The W425R and L443F FBXW7 mutants can specifically induce the degradation of MCL1, but not other targets [86]. These observations suggest that mutations in FBXW7 may introduce a selective degradation pattern of target proteins, which is associated with the genetic background present in each tumor cell(s). This further suggests that certain FBXW7 targeted oncogenes, may be more necessary in individual cancers, than others. This would then suggest why certain cancers have a propensity for a particular FBXW7 mutation, in which certain pro-survival functions are required. Finally, recent studies have reported gain of function mutations for FBXW7. The FBXW7 mutants D510E and D527G were found to only degrade NOTCH1 [87]. Surprisingly, these mutants were associated with an oncogenic phenotype and cooperated with viral and/or cellular oncogenes in colony formation assays and *in vivo* tumor growth [87]. This functional difference between FBXW7 mutants implies that future research on FBXW7 mutations should be directed towards a more in-depth and refined course. It may be necessary to differentiate mutation types and functions of different mutants to clearly distinguish between fully inactivating mutations and those with partially inactivating mutations. These studies will have important implications for cancer treatment by targeting appropriate signaling pathways activated in tumor cells.

**Fig. 5:**
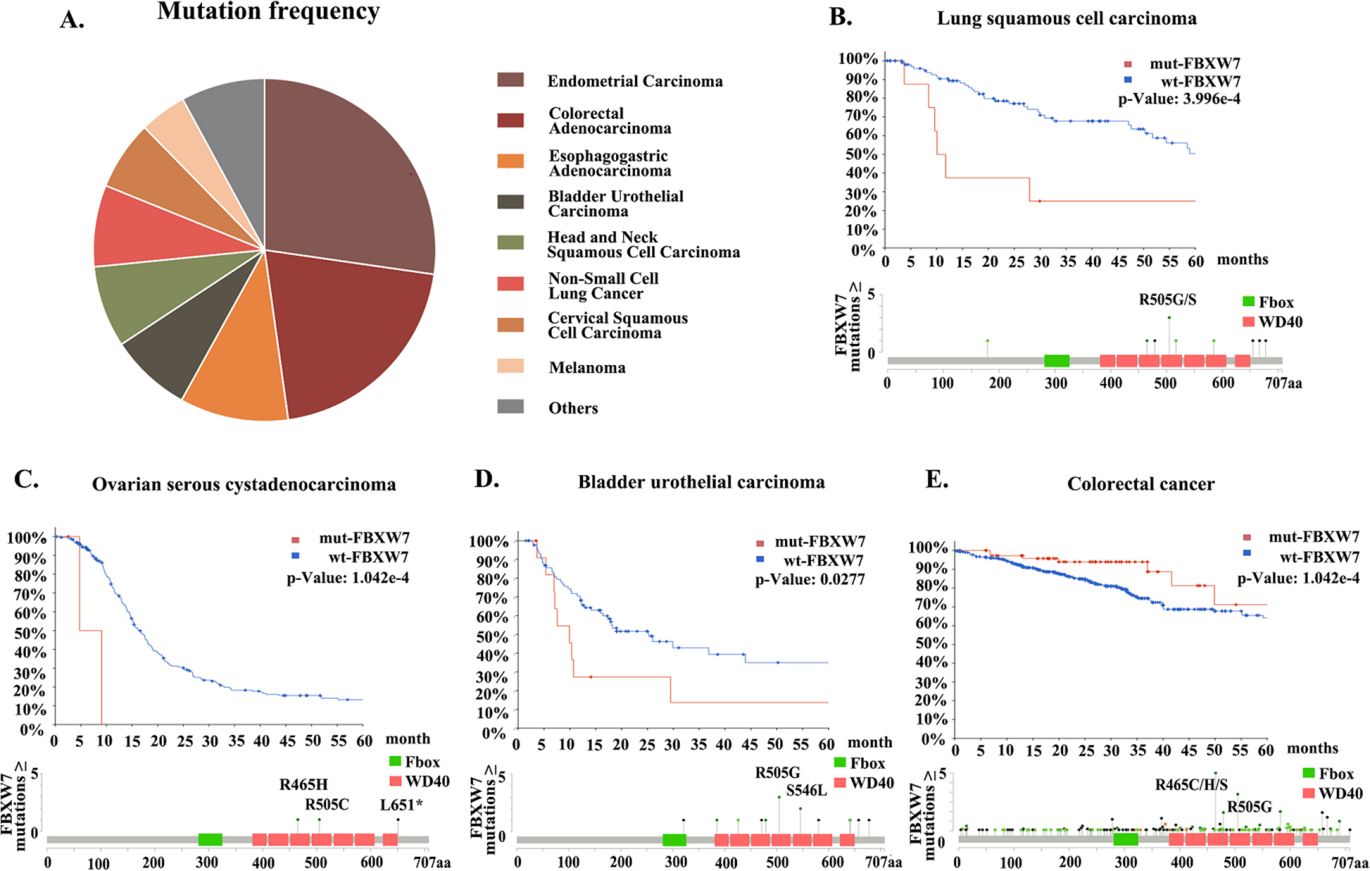
The Contribution of FBXW7 Genetic Mutations to Cancer Type and Progression. FBXW7 loss or mutation in prognosis and cancer progression. **A** The cBioportal database statistics on the incidence of FBXW7 mutations in selected tumors from relevant clinical trial information (https://www.MYCancergenome.org/). **B-E** Kaplan-Meier survival analysis and mutation point is performed using cBioportal on TCGA database related datasets to obtain disease-free survival curves for patients with squamous cell carcinoma of the lung, ovarian plasmacytoma, and urothelial carcinoma of the bladder (http://www.cbioportal.org/)

FBXW7 shows distinct mutational probabilities in different cancers. According to the clinical information related to FBXW7 in the cBioportal database, of the TCGA Pan Cancer dataset, FBXW7 has mutations in a wide variety of cancers (Fig. 5A). Mutations within FBXW7 can mediate the poor prognosis of tumor therapy through multiple pathways. In colorectal cancer, mutations of FBXW7 can lead to immune escape from lytic T-cell immunosurveillance. This results in poor survival in colorectal cancer patients [88]. Similar observations have been made in studies related to mesothelioma [89]. In lung squamous cell carcinoma, mutations in the FBXW7 gene have been observed to affect survival in experimental animal models [90]. Lung squamous cell carcinoma (cBioportal analysis, TCGA, Firehose Legacy) datasets show that mutations in FBXW7 significantly affect patient survival (Fig. 5B). This correlation between FBXW7 mutations and disease prognosis is even more explicit for ovarian serous carcinoma; whereby FBXW7 can be used as an independent prognostic indicator [91]. This association was further validated by the cBioportal for the TCGA ovarian serous cystadenocarcinoma dataset (TCGA, Nature 2011), and the results showed that FBXW7 mutations may be an important factor affecting ovarian serous carcinoma (Fig. 5C). Next generation sequencing also identified FBXW7 as a potential therapeutic target for bladder cancer [92], which was validated in bladder uroepithelial carcinoma datasets (cBioportal, TCGA, Firehose Legacy) (Fig. 5D). In a comprehensive analysis of multiple data sets on colorectal cancer, it was also verified that mutations in FBXW7 mediated poor prognosis in colorectal cancer (Fig. 5E), which is consistent with the close association of FBXW7 mutations with colorectal cancer mentioned previously. Besides solid tumors, FBXW7 mutations also have an adverse effect on survival in adult T-cell leukemia (ATL) cells [86]. In addition, several T-cell leukemia (T-ALL)-related studies have indicated that mutations in FBXW7 are associated with poor prognosis in T-ALL [93–96].

#### Clinical significance of FBXW7 mutations

There is a strong association between inactivation of FBXW7 and the development of drug resistance in the treatment of tumors [90, 97]. However, there are differences in the extent to which FBXW7 mutations affect the drug resistance of different therapeutic agents. The Genomics of Drug Sensitivity in Cancer (GDSC) database can expose the association between FBXW7 mutations and drug sensitivity through ANOVA analysis. In fact, such analysis of the GDSC data shows that FBXW7 mutations have a particularly significant effect on the sensitivity of therapeutic drugs (Table 3). Although loss of FBXW7 is relevant to most human cancers, this section will focus on those most frequently associated cancers with FBXW7 mutations.

**Table 3:** Effect of FBXW7 mutations on drug treatment sensitivity

Drug	Drug Target	IC50		*P*-value	
		mut-FBXW7	wt-FBXW7		
UNC0638	G9a and GLP methyltransferases	24.909	19.849	0.00999	
KIN001-266	MAP3K8	32.119	20.622	0.0265	
Temsirolimus	MTOR	0.3013	0.13204	0.0244	
BIX02189	MEK5, ERK5	97.184	79.736	0.0232	
Tubastatin A	HDAC1, HDAC6, HDAC8	149.28	108.77	0.0254	
Shikonin	not defined	1.4784	0.94584	0.0109	
KIN001-042	GSK3B	114.01	89.295	0.0477	
THZ-2-49	CDK9	36.568	11.993	0.0246	
KIN001-042	GSK3B	114.01	89.295	0.0289	
JAK1_3715	JAK1	109.83	79.93	0.0394	
Tanespimycin	HSP90	0.93356	0.45133	0.0261	
OSI-930	KIT	77.599	60.839	0.0325	
AICA Ribonucleotide	AMPK agonist	3268.4	2217.1	0.0349	
Venetoclax	BCL2	9.8217	9.1615	0.0209	
WEHI-539	BCL-XL	35.43	34.404	0.0263	

This table list drugs with resistance caused by FBXW7 mutations adapted from the GDSC database (https://www.cancerrxgene.org/). The name of the drug is listed along with the pathway targeted. Only data with *P* values of less than 0.05 were considered

Colorectal carcinoma (CRC) progression demonstrates a strong negative correlation with FBXW7 expression [98]. Recent genome sequencing studies suggest that as much as 16% of CRC patients demonstrate mutation(s) in FBXW7 [99]. In metastatic CRC patients the loss of FBXW7 is associated with shorter overall survival (OS) and drug resistance [100]. In a retrospective CRC study, comparing cancer tissues and their paired adjacent non-cancerous tissues, FBXW7 expression was negatively associated with lymph node metastasis and TNM stages [101]. A meta-analysis involving 4199 patients predicted poor OS but no significant change in disease-free survival (DFS) in CRC patient with loss of FBXW7 [102]. Furthermore, multivariate Cox regression analyses from several studies suggests that FBXW7 expression is an independent prognostic biomarker of poorer outcome for CRC patients [103, 104]. These observations are corroborated by preclinical and *in vitro* studies. Numerous *in vitro* studies from multiple laboratories have demonstrated that loss of FBXW7 in colon cancer cells is significantly associated with resistance to drug therapies. These treatments included, PD-1 antibody, Taxol, Oxaliplatin, RAS/RAF/MEK/ERK signaling multi-kinase inhibitors, and irinotecan (CPT-11) [105–109]. These results demonstrate significant clinical challenges posed by the loss of FBXW7 expression in tumor cells.

Endometrial cancers (ECs) are aggressive tumors that frequently harbor somatic missense mutations in FBXW7 [110]. FBXW7 mutations are found in up to 30% of serous ECs, 28% of uterine carcinosarcomas, 25% of clear cell ECs, and 10% of endometrioid ECs. Studies have demonstrated that loss of the phosphatase, PP2A (Protein Phosphatase 2 Phosphatase Activator) or FBXW7 constitute drivers in EC progression [111, 112]. Furthermore, FBWX7 mutations were strongly associated with late-stage, vascular invasion, and lymph node metastasis [113]. These findings and related studies suggest that FBXW7 deficiency mediates poor progression of EC; and is associated with resistance problems related to therapies including PD-1 inhibitors [105].

The expression of FBXW7 shows a significant correlation with the treatment outcome of non-small cell lung cancer (NSCLC) [114–116]. Whole-exome sequencing and comprehensive immunoassays of early-stage lung squamous carcinoma reveals a significant decrease in FBXW7 expression levels, suggesting that FBXW7 may be one of the key factors influencing the progression of lung squamous carcinoma [115]. Cancer Genome Atlas data suggests that deletion of FBXW7 occurs in 31% of lung adenocarcinomas (LADC) and 63% of lung squamous cell carcinomas (LSCC). Loss of FBXW7 promotes the development and progression of LSCC and LADC through activation of KRAS and contributes to hyperactivation of NF-κB, which increase resistance to cisplatin treatment [90] as well as anti-EGFR therapies such as Gefitinib, Cetuximab, or Panitumumab. Finally, additional studies show that hyperactivation of ERK1/2 signaling decreases FBXW7 protein stability and prevents degradation of phosphorylated HSF1 (Heat Shock Transcription Factor 1). This in turn, stimulates transcription of multi-drug resistance receptor (MDR1) gene and drug resistance, which affects the treatment of NSCLC.

A high mutation rate of FBXW7 has been reported in hematopoietic cancers, with 20% of T-cell non-Hodgkin lymphoma (T-NHL), 14% of T-cell acute lymphoblastic leukemia (T-ALL) and 10% of HTLV-I-associated acute T-cell leukemia (ATL) patient’s cells harboring FBXW7 mutations. In a preclinical study, FBXW7 mutations R505C, R465H, R465C, or R479Q conferred resistance to gamma secretase inhibitor (GSI), MRK-300, in T-ALL. The underlying resistance mechanism was found to be related to the inability of mutated FBXW7 to target NOTCH and c-MYC for proteasome degradation [117]. In a preclinical study, adult T cell leukemia cells expressing FBXW7 mutants W425R, S462P, and H468R demonstrated resistance to BET inhibitors JQ1 and Birabresib (OTX015). This was associated with increased phosphorylation of BRAF, MEK, and ERK, and increased expression of Stat3 and MYC.

FBXW7 mutations have also been detected with a rate of 8% in melanoma patients [118]. Like CRC cells, in a preclinical study, loss of FBXW7 was associated with resistance to immunotherapy treatment with PD-1 antibody [119]. In an additional clinical case study, a melanoma patient treated with Keytruda (Pembrolizumab) demonstrated a response in all skin lesions but one [105]. Genome sequencing revealed that the lesion resistant to Keytruda was mutated for FBXW7 at R505C.

#### Post-transcriptional regulation of FBXW7

FBXW7 expression is also regulated post-transcriptionally at the protein level. FBXW7 can be phosphorylated by several proteins, leading to loss of dimerization and subsequent degradation. Among the proteins phosphorylating FBXW7 are ERK1/2 (Mitogen-Activated Protein Kinase), PLK1/2 (Polo-like kinase-1/-2), and CDK5 (cyclin dependent kinase 5). FBXW7 can interact with ERK kinases, resulting in the phosphorylation of FBXW7 at Thr205, which triggers binding to Pin1 (Peptidyl-prolyl cis-trans isomerase NIMA-interacting 1) [120]. Pin1 is known to isomerize specific Ser/Thr-Pro peptide bonds and to regulate their conformational changes [121]. Following binding, Pin1 prevents dimerization of FBXW7, encouraging self-ubiquitination and degradation [5]. A similar method is carried out by PLK proteins. PLK-1 directly phosphorylates FBXW7, leading to self-ubiquitination and degradation. This sets up a regulatory loop, since PLK1 can be transcriptionally activated by c-MYC and N-MYC, which are both degradation targets of FBXW7 [122, 123]. Another PLK family member, PLK2, has also been shown to phosphorylate FBXW7, which can lead to uncontrolled centriole duplication [123]. CDK5 is an additional kinase which can negatively regulate FBXW7 through phosphorylation and degradation [124]. Studies have also shown a separate role for CDK5 in priming the microtubule organization protein, NDE1 (NudE Neurodevelopment Protein 1) for degradation by FBXW7 [125]. Altogether these feedback regulatory loops point to a dynamic back and forth between FBXW7 expression, FBXW7 degradation, and the amount of FBXW7 substrates. Not all FBXW7 degradation is mediated through protein phosphorylation. The histone demethylase, LSD1(lysine-specific demethylase 1), also plays a role in controlling FBXW7 activity. Unlike the proteins listed above, which phosphorylate FBXW7, LSD1 binding promotes FBXW7 self-ubiquitylation, leading to both proteasomal and lysosomal degradation of FBXW7 (Fig. 1) [126]. FBXW7 can also be inactivated through mislocalization. Protein kinase C (PKC) specifically phosphorylates the α-isoform of FBXW7, which regulates its nuclear localization [127].

FBXW7 protein stability is affected by a balance between ubiquitination (K404 and K412) and interactions with deubiquitinating enzymes (DUB). TRIM25 (tripartite motif-containing 25) is an E3 ligase, which has been shown to ubiquitinate FBXW7 at K412 triggering its proteasomal degradation [128]. However, recent studies suggest that K412 is not sufficient for FBXW7 degradation and that additional TRIP12-mediated branched K11 is required for FBXW7 degradation [129]. DUBs are classical regulators of FBXW7 [130, 131]. One such protease, USP28, (Fig. 1) can bind to multiple structural domains of FBXW7 to inactivate FBXW7 auto-ubiquitination as well as FBXW7-mediated ubiquitination of various targets [132, 133]. USP28 acts in the nucleoplasm and reverses FBXW7α-, but not FBXW7γ-mediated MYC ubiquitination and degradation [132, 133]. In contrast, USP36 promoted de-ubiquitination of MYC in the nucleolus by both FBXW7 isoforms and increased MYC stability [134] (Fig. 1). An additional DUB, USP9X, has been shown to act as a positive regulator of FBXW7 stability by preventing its ubiquitination and degradation. Expectedly, loss of USP9X was associated with poor prognosis in colorectal cancer [135].

A different strategy to inactivate FBXW7 functions relies upon the disruption of the FBXW7/target SCF complex. Some proteins have been shown to interact with FBXW7 while complexed with or without its substrate(s) and therefore prevents subsequent substrate degradation. This is the case for the pseudophosphatase STYX, which binds to the F-box domain of FBXW7 [136]. Without recruitment to the SCF complex, FBXW7 is no longer able to target its substrates for ubiquitination. There are many other factors that can interact with FBXW7 directly or indirectly to affect FBXW7 downstream functions. Among these factors are CSN5 and 6, RACK1, PI3K, EBP2, and PKC. CSN6 (COP9 signalosome subunit 6) is part of a cycle with CSN5, FBXW7, and cullin proteins with the ratio of CSN5 and 6 causing degradations of FBXW7. For example, CSN6 can associate with FBXW7, which allows for autoubiquitination of FBXW7 and efficiently prevents ubiquitination of FBXW7 targets [137]. Other factors, such as RACK1 (Receptor for Activated C Kinase 1) can act as adaptor proteins for substrate interaction(s) with FBXW7. RACK1 can bind non-phosphorylated c-Jun and complex it with FBXW7, marking c-Jun for degradation. However, RACK1 cannot recruit phosphorylated c-Jun to FBXW7. Interestingly, this efficiently makes phospho-c-Jun resistant to FBXW7-mediated degradation [138]. In contrast, PI3K-dependent phosphorylation of FBXW7 has been proposed as a fine-tuning mechanism that stabilizes FBXW7 [139]. Sequestration of FBXW7 away from its targets has also been reported. Both EBP2, a pseudo substrate of FBXW7, and PKC redirect cellular localization of FBXW7 proteins to the nucleolar compartment preventing its access to other substrate(s) [127, 140].

### Oncogenic signaling pathways regulated by FBXW7

#### Cell cycle checkpoints and tumor cell proliferation

FBXW7-mediated ubiquitination and proteasome degradation control the expression of many proteins with key functions in the regulation of cell cycle progression [117] (Table 4). Many checkpoints are activated in response to stimuli such as DNA damaging agents to prevent errors from being replicated and to allow the cell to arrest and repair damages or undergo cell death. Loss of FBXW7 releases these safeguards, facilitating tumor cell proliferation and accumulation of genetic defects. Some important proteins controlling the G1/S transition of the cell cycle are regulated by FBXW7. These include c-MYC, Cyclin E and p53 [141–143]. c-MYC is generally associated with an increase in the activities of cyclin-dependent kinases, Cdk2, Cdk4 and Cdk6, as well as inhibition of p21WAF/CIP and p27KIP. Thus, inhibition of FBXW7 allows rapid unchecked progression through the G1 to S phase of the cell cycle [144]. The CDK2/Cyclin E complex regulates cell cycle progression by licensing DNA replication at the G1-S phase transition [145]. The inactivation of FBXW7 leads to high levels of c-MYC and Cyclin E and the inability of cells to withdraw from the cell cycle leading to uncontrolled proliferation. Several laboratories have also reported that FBXW7 interacts with and targets p53 for ubiquitination and proteasomal degradation [146–148]. In response to DNA damage, the serine/threonine kinase, ATM stimulates p53 phosphorylation at Ser15. This allows for stabilization of p53, release of Mdm2, and increased transcription of genes involved in cell cycle arrest. Additional phosphorylation by DNA-PK and GSK3β promotes FBXW7-mediated degradation of p53 to allow the cell cycle to resume after DNA damage has been repaired [149]. Although it is unclear at this time how FBXW7-mediated regulation of p53 may participate in tumorigenesis it is noteworthy that experimental deletion of FBXW7 in murine models of breast cancer is linked to the acquisition of p53 mutations. In concordance, database analyses revealed the co-occurrence of FBXW7 disruption and p53 mutation(s) in breast cancer [150], gastric cancer [151] and high grade serous ovarian cancer [152].

**Table 4:** Targets of FBXW7

Factor	Description	Expression	References
SKP1	S-phase kinase-associated protein 1	↓	[136, 153–165]
		↑	[135, 155, 157, 158]
MYC	v-myc avian myelocytomatosis viral oncogene homolog	↓	[166]
		↑	[155, 157, 158]
CUL1	cullin 1	↓	[136, 153, 154, 158, 163, 165, 167–173]
		↑	[155, 157, 158, 174–177]
CCNE1	cyclin E1	↓	[126, 162, 163, 165, 172, 178–188]
NOTCH1	NOTCH 1	↓	[126, 163, 164, 180, 189–192]
		↑	[157, 193]
FBXW7	F-box and WD repeat domain containing 7, E3 ubiquitin protein ligase	↓	[5, 126, 136, 181, 187, 189]
		↑	[135, 157]
JUN	jun proto-oncogene	↓	[188, 191, 194, 195]
		↑	[135]
MCL1	myeloid cell leukemia 1	↓	[5, 153, 196, 197]
SREBF1	sterol regulatory element binding transcription factor 1	↓	[187, 198, 199]
RBX1	ring-box 1, E3 ubiquitin protein ligase	↓	[153, 163, 165]
		↑	[157, 176]
STYX	serine/threonine/tyrosine interacting protein	↓	[136, 200]
		↑	[136, 201]
MED13	mediator complex subunit 13	↓	[169]
		↑	[155, 157]
KLF5	Kruppel-like factor 5 (intestinal)	↓	[202]
MED13L	mediator complex subunit 13-like	↓	[155]
		↑	[157, 169]
TP53	tumor protein p53	↓	[146]
		↑	[175, 176]
MYCBP2	MYC binding protein 2, E3 ubiquitin protein ligase	↓	[178]
		↑	[135, 155, 157, 175, 176]
NFKB2	nuclear factor of kappa light polypeptide gene enhancer in B-cells 2 (p49/p100)	↓	[163, 203]
		↑	[155, 158, 204]
FBXO45	F-box protein 45	↓	[157]
		↑	[155, 157, 175, 176]
MTOR	mechanistic target of rapamycin (serine/threonine kinase)	↓	[191, 205]
		↑	[135, 157]
AURKA	aurora kinase A	↓	[181, 191, 206]
HSF1	heat shock transcription factor 1	↓	[155, 207, 208]
		↑	[155]
BLM	Bloom syndrome, RecQ helicase-like	↓	[194]

List of the most common, experimentally verified, FBXW7 targets degraded by ubiquitination (https://thebiogrid.org). According to the available literature, FBXW7 can either up- or down-regulate the expression of the listed target proteins. Some cellular targets can be up-regulated and down-regulated in different cancer types or under distinct physiological conditions. References for studies which describe target degradation is listed on the right-end side

#### DNA damage repair and Genome instability

DNA-damage checkpoint kinase, PLK1 (Polo like kinase-1) plays an essential role in the regulation of DNA replication, regulating pre-replicative complex formation by phosphorylating several proteins [209]. Studies show that FBXW7 can target PLK1 for degradation in response to DNA double strand breaks (DSB) [210]. This event allows transient cell cycle arrest and participation of FBXW7 in DNA repair. Several studies have reported that in response to DSB, FBXW7 binds to PARP (Poly (ADP-Ribose) Polymerase 1) and is rapidly recruited to DNA damage sites [211]. In response to DSBs, ATM is activated and induces phosphorylation of FBXW7 at Ser26 [212]. This promotes the retention of FBXW7 at DSBs, where it interacts with the DNA repair protein, XRCC4 (X-Ray Repair Cross Complementing 4). Unlike traditional targets of FBXW7, XRCC4 is K63 ubiquitinated, an event that facilitates the recruitment of KU80/70 and the initiation of non-homologous end joining (NHEJ) DNA damage repair [212]. FBXW7 has also been involved in other types of DNA repair by regulating degradation of FAAP20, a critical component of the Fanconi anemia (FA) DNA interstrand cross-link (ICL) repair pathway [213]. In addition to DNA repair pathways, FBXW7 plays critical roles during mitosis by acting on multiple targets to ensure proper chromosome segregation and genome stability [214, 215]. Overexpression of Cyclin E, AURKA or PLK1 following the loss of FBXW7 is associated with centrosome duplication and the formation of pseudo-bipolar or multipolar mitotic spindles. The latter is strongly associated with chromosome missegregation, genome instability and aneuploidy. During M phase, AURKA targets a multitude of proteins and plays critical roles in regulating mitotic spindle assembly (SAC), centrosome duplication, chromosome segregation, and cytokinesis. Thus, de-regulation of AURKA, through FBXW7, has implications in genomic stability [206]. As stated above, the kinase, PLK2, can phosphorylates FBXW7 to decrease the stability of FBXW7 [123]. This allows a transient increase of Cyclin E that is essential for centriole reproduction. In contrast, loss of FBXW7 leads to overexpression of Cyclin E and uncontrolled centriole duplication. FBXW7 also controls the proteolysis of Cyclin E to prevent CENP-A hyperphosphorylation and licenses normal assembly of CENP-A during mitosis. Thus, loss of FBXW7 increases Cyclin E/CDK2-mediated phosphorylation of CENP-A at Ser18 and stimulates chromosomal abnormalities [216]. Furthermore, FBXW7 targets the centriole assembly protein HsSAS6; and FBXW7 inactivation increases HsSAS-6 expression and its recruitment at multiple sites to the mother centriole, leading to supernumerary centrioles [217]. During M phase, AURKA targets a multitude of proteins and plays critical roles in regulating mitotic spindle assembly (SAC), centrosome duplication, chromosomes segregation and cytokinesis [218]. All these studies clearly demonstrate why loss of FBXW7 has far-reaching implications for unchecked proliferation of tumor cells, chromosome instability, and carcinogenesis.

#### Regulation of cell survival

Consistent with its tumor suppressor functions FBXW7 is generally an inducer of apoptosis, and its loss of function promotes increased survival and resistance to apoptotic signals. FBXW7 promotes ubiquitination and degradation of myeloid leukemia 1 (MCL-1) a member of the pro-survival Bcl-2 family regulating apoptosis in tumor cells [219, 220]. MCL-1 has a high affinity for pro-apoptotic Bcl-2 family members, Bax, Bak, Bim, Bid, Puma and Noxa compared to other Bcl-2 pro-survival family members, suggesting that MCL-1 is an essential sentinel against pro-apoptotic signals. Upregulated expression of MCL-1 following the loss of FBXW7 has been described in many cancer types and shown to correlate with increased resistance of tumor cells to various therapies. FBXW7 also regulates YAP (Yes-associated protein), a negatively regulated downstream effector of the Hippo signaling pathway that plays an important role in inhibiting apoptosis [221]. Clinical studies have demonstrated that YAP is overexpressed in tumors and associated with poor survival due to resistance to apoptosis [222]. Consistent with the fact that FBXW7 targets YAP for degradation [223], a direct correlation between the loss of FBXW7 and high expression of YAP has been demonstrated in HCC, gastric cancer (GC), and pancreatic cancers [224–226]. In addition, increased expression of YAP can stimulate transcription of SOX9 a transcription factor degraded by FBXW7 in response to DNA damage caused by cytotoxic drugs or UV-irradiation [227]. In the absence of FBXW7, high levels of SOX9 favor cell survival and accumulation of genetic defects [228]. FBXW7 also plays roles in other forms of cellular death. Ferroptosis is an iron-dependent form of non-apoptotic cell death, which is triggered by the oncogenic RAS selective lethal small molecule erastin [117, 229]. FBXW7 can trigger ferroptosis by inhibiting the expression of stearoyl coenzyme A desaturase (SCD1) through the inhibition of nuclear receptor subfamily 4 group A member 1 (NR4A1), which regulates lipid peroxidation and promotes ferroptosis [120, 230].

#### Role of FBXW7 in the tumor microenvironment and tumor immunity

Tumor cells can functionally shape the immune microenvironment required for their proliferation [231]. In cancer, FBXW7 can inhibit macrophage M2-type polarization by mediating c-MYC degradation. M2-type macrophages are a pro-oncogenic phenotype that promotes angiogenesis by enhancing the invasive properties of tumor cells, remodeling the extracellular invasive matrix, and favoring tumor cell proliferation. FBXW7 inhibits the direction of macrophage M2-type polarization by inhibiting the tumor immune microenvironment thereby affecting cancer progression [232]. FBXW7 also modulates the tumor immune microenvironment by mediating the degradation of C/EBPδ, thereby inhibiting Toll-like receptor 4 expression, reducing inflammation, and modulating the innate immune response of macrophages to pathogens [233]. The tumor microenvironment can also be altered through the process of epithelial mesenchymal transition (EMT), a process by which epithelial cells are transformed into migratory and invasive cells [234]. EMT leads to the spread of individual cancer cells from the primary tumor site, participates in the dedifferentiation process leading to malignant cancer, and increases the malignancy of the tumor [235]. Several proteins regulated by FBXW7 influence EMT such as EMT transcription factors (EMT-TFs), including mTOR, Snai1, c-MYC [236, 237], SOX9 [227, 238], and ZEB2 [100]. Studies have also shown that CCL2 (C-C Motif Chemokine Ligand 2) activation promotes metastatic ecotone formation through recruitment of monocytic myeloid-derived suppressor cells and macrophages [239, 240]. FBXW7 can inhibit CCL2 gene transcriptional activation by negatively regulating NOTCH expression levels; and inhibition of CCL2/CCR2 signaling has been shown to suppress tumor metastasis demonstrating an important role of the FBXW7/NOTCH/CCL2 axis in the regulation of cancer metastasis [241]. In addition, FBXW7 was found to control CCL2 expression by stimulating ubiquitination of EZH2 (enhancer of zeste homolog 2); thereby reducing global H3K27me3 on the promoter of CCL2 [242]. FBXW7 has also been shown to control innate immunity signaling pathways. Loss of FBXW7 has been associated with a reduced ability of cells to activate double strand RNA (dsRNA) and interferon (IFN) responses normally activated in cells infected with tumor RNA viruses. Impairment in sensing dsRNA in FBXW7 deficient cells was associated with a loss of MDA5 (Interferon Induced with Helicase C Domain 1) and RIG-I (Retinoic Acid-Inducible Gene 1 Protein) expression and associated with resistance to anti-PD-1 therapies [105]. In addition, loss of FBXW7-mediated proteasomal degradation of IFN-γ receptor 1 (IFNGR1) results in the constitutive activation of IFNGR1 signaling. Activated IFNGR1 stimulates the recruitment of immune suppressive neutrophils to the tumor site, facilitating tumor growth and metastasis formation in patients with triple negative breast cancer (TNBC) [243].

#### Targeting FBXW7: Development of novel therapeutic strategies.

As detailed above, activation of specific signaling pathways that promote tumor cell proliferation, cell cycle progression, or oncogenesis, such as Erk1/2, Pin1 and PLK1, have been associated with FBXW7 auto-ubiquitination and degradation. In turn, loss of FBXW7 expression is associated with a reduced turnover of downstream targets. This results in the accumulation of specific oncoproteins and oncoprotein dependency that can create an opportunity for targeted therapies in tumor cells. Several strategies are worth pursuing in targeting cancers dependent on FBXW7 loss of function(s): 1- targeting the reactivation of FBXW7 expression in the case of tumors with wild type FBXW7 that is inactivated through epigenetic mechanisms, or 2-targeting downstream effectors of FBXW7 inactivation in case of gene mutation or deletion.

#### Restoration of FBXW7 tumor suppressor functions to increase drug sensitivity

Overexpression of wild-type FBXW7 can restore the ability to ubiquitinate target proteins in cells not expressing FBXW7. This suggests that the drug resistance problem caused by FBXW7 loss may be reversible. As discussed previously, numerous studies have demonstrated the clinical significance of FBXW7 promoter hypermethylation in carcinogenesis [20–22]. Inhibition of DNA methyltransferases by decitabine has been used in various preclinical and clinical settings with promising results. Regarding FBXW7, a recent study showed that both, *in vitro* lung cancer cells and *in vivo* lung cancer xenograft mouse models treated with decitabine revert the FBXW7 promoter to an unmethylated state. This resulted in increased expression of FBXW7 followed by decreased expression of MCL-1. Consequently, use of the Bcl-2 inhibitor, Venetoclax, and decitabine demonstrated strong synergistic toxicity against lung cancer cells [244]. NSCLC cells with low expression of FBXW7 are resistant to microtubule targeting agents including Taxol. However, NSCLC cells with loss of FBXW7 are highly sensitive to class 1 histone deacetylase (HDAC) inhibitor, Entinostat MS-275. In addition, Taxol resistance was effectively overcome by MS-275 treatment, suggesting that HDAC inhibitors may have therapeutic benefits in the treatment of aggressive Taxol-resistant NSCLCs that have loss of FBXW7 expression [245]. Similar results were also published on hematological cancer cells harboring FBXW7 inactivating mutations [246]. In a preclinical study, the histone deacetylase inhibitor, Beleodaq (belinostat) also inhibited human hematologic cancer cell lines harboring FBXW7 inactivating mutations in culture [247]. The downfall of using these therapies to treat cancer, is that DNA methyltransferases drugs have a global impact on cellular gene expression. While FBXW7 has the potential to be re-activated, so would addition hypermethylated genes, which may include repressed oncogenes. This could potentially be overcome if FBXW7 could be specifically targeted with gene editing technology.

Another route to restore FBXW7 expression may be to target FBXW7-microRNAs in tumor cells that harbor wild-type FBXW7 mRNA. Studies performed *in vitro* and in animal models have shown several promising approaches including LNA-antimiRs or small molecule inhibitors of miRNAs (SMIR), which prevent miRNA-target interaction(s). Some of these are developed by Regulus Therapeutics and are currently in clinical trials for the treatment of HCV cancer [248]. One potential issue may be related to the unintended side effect of miRNAs, since each miRNA has dozens of gene targets. With the development of quantum computing, highly complex networks may be solved and offer clarity for the best approach to inactivate selected miRNAs in cancers.

In some instances, cancers cells express low levels of FBXW7 protein because of pathologic rapid turnover. This can readily be observed in cells with hyperactivation of ERK1/2 signaling or high activity of PLK1 or Pin1. Specific small inhibitors for these pathways can restore FBXW7 expression and/or induce cell cycle arrest or apoptosis. These inhibitors, PLK1 inhibitor onvansertib and Pin1 inhibitor sulfopin, are currently being tested in clinical trials [249, 250]. While there is no specific ERK1 inhibitor currently approved by the FDA, there are several inhibitors targeting the upstream kinase MEK1 (Benimetinib, Cobimetinib, Selumetinib and Tremetinib). In addition to FBXW7 self-ubiquitination, studies have shown that in melanoma cells the nuclear accumulation of MDM2 leads to MDM2-dependent ubiquitination and rapid downregulation of FBXW7 resulting in upregulation of FBXW7-degraded oncoproteins such as p63 [251, 252]. Although melanoma cells are highly resistant to MAPK inhibitors, a combination with MDM2 inhibitor, Nutlin-3A, resulted in elevated FBXW7 expression, p63 degradation, and apoptosis of melanoma cells effectively reversing MAPK inhibitor resistance [253].

#### Targeting downstream effectors activated in FBXW7 negative tumors

Several studies have shown that deletion of FBXW7 can lead to targeted therapy resistance by stabilizing MCL-1 [153, 254]. Heat shock protein 90 (Hsp90) is an oncoprotein that is widely expressed in cancers and acts to promote malignant progression of tumors that can be inhibited by Hsp90 inhibitors [255, 256]. However, the sensitivity of Hsp90 inhibitors is limited by the level of MCL-1, which is overexpressed in some cancers due to FBXW7 mutations [107]. This makes the efficacy of Hsp90 inhibitors limited. However, the sensitivity of cancer cells to Hsp90 inhibitors can be restored following inhibition of MCL-1 [257, 258]. In this regard, FBXW7 mutations could be proposed as resistance markers for Hsp90 inhibitor targeting [259]. Somatic FBXW7 mutations in squamous cell carcinoma (SCC) are associated with stabilized MCL-1 and high Bim levels [260]. This results in a poor response to standard chemotherapy but a robust response to HDAC inhibitors and enhanced synergy with the combination Vorinostat/ABT-737. As described above, PLK1 stimulates phosphorylation and degradation of FBXW7. The use of PLK1 inhibitors to induce toxicity in tumor cells with loss of FBXW7 has been reported in many studies. Investigations demonstrate that a combination of PLK1 (BI2536) and Bcl-2 (ABT199) inhibitors synergized and induced apoptosis in FBXW7 low, and c-MYC overexpressing, tumor cells [122]. Several AURKA inhibitors, another target of FBXW7, have also shown promising results *in vitro* and in preclinical studies and several inhibitors have progressed to phase II/III clinical trials [261, 262].

#### Targeting tumors carrying mutated inactive FBXW7

To date, there are no reports of any specific drugs directly targeting FBXW7. DrugBank uses the DNA sequence of FBXW7 as a starting point, applying the target-drug match-related function of FBXW7, which allows querying the potentially effective inducers of FBXW7 (Table 5). The DrugBank platform showed that there are 14 drugs that may bind to bases 148878 to 148938 of FBXW7, which can act as an inducer with FBXW7. Whether these drugs may be effective in targeting cells with loss of FBXW7 remains to be investigated.

**Table 5:** FBXW7 potential inducers

Combining regions	Drug	Drug Research PhaseDrug Investigation Phase	Action
148878-148938	Troleandomycin	approved	inducer
	Dexamethasone	approved, investigational, vet_approved	inducer
	Rifampicin	approved	inducer
	Midostaurin	approved, investigational	inducer
	Dexamethasone acetate	approved, investigational, vet_approved	inducer
	Myrrh	approved	inducer
	Brigatinib	approved, investigational	inducer
	Rifapentine	approved, investigational	inducer
	Prednisone	approved, vet_approved	inducer
	Methylprednisolone	approved, vet_approved	inducer
	Lemborexant	approved, investigational	inhibitor/inducer
	Prednisolone phosphate	approved, vet_approved	inducer
	Relugolix	approved, investigational	inducer
	Ursodeoxycholic acid	approved, investigational	inducer

Potential inducers for FBXW7 based on DrugBank database prediction tool, screened based on the sequences of FBXW7 in GeneBank. According to the database, “The term “inducer” can be a drug or chemical that typically serves to induce or trigger the appropriate gene transcription necessary to synthesize new copies of a particular enzyme, in addition to what quantity may already be present for that enzyme in the body” (https://www.drugbank.com/)

There is limited data on studies aimed at targeting cells with mutated FBXW7. Metastatic unresectable malignant melanoma (MM) is inherently characterized by poor radiotherapy results. The combination of FDA-approved mTOR inhibitors, Everolimus and Temozolomide, has significant therapeutic effects and improved survival in advanced MM patients carrying FBXW7 mutations [263]. In xenograft mouse models of lung cancer, cells harboring FBXW7 R465H were particularly sensitive to treatment with the mTOR inhibitor, Torisel (temsirolimus) [264]. A preclinical study also showed that breast cancer cells with mutated FBXW7 have increased sensitivity to the mTOR inhibitor, Rapamune (Sirolimus) [265]; and phase 1 studies demonstrated prolonged and stable disease in a patient with hepatocellular fibrolamellar carcinoma harboring FBXW7 E192A treated with Rapamune [266]. Tumor cell lines with deletions or mutations in FBXW7 were particularly sensitive to rapamycin treatment suggesting that loss of FBXW7 may serve as biomarker for an increased susceptibility to inhibitors of the mTOR pathway [267]. A study reported that FBXW7-associated drug resistance is reversed by induction of terminal differentiation in murine intestinal organoid culture. Further results support the hypothesis that a differentiating therapy may successfully target FBXW7-mutated CRC cells [265]. However, it appears that no follow up studies have been published in the area.

Tumor mutation profiling can reveal co-occurring mutations and an opportunity to distinguish between favorable and unfavorable gene profiles in response to specific chemotherapy or targeted therapies ensuring maximum patient benefits from treatment [268]. In a clinical study of therapies for solid tumors with PIK3CA mutations, in which co-occurring mutations such as the FBXW7 mutation were also detected, the investigators uniquely found that Alpelisib (BYL719) selectively inhibited PI3Kα in solid tumors with PIK3CA mutations containing the FBXW7 co-occurring mutation, effectively inhibiting tumor growth and improving patient survival [269]. In another clinical trial, LY3039478 treatment resulted in a partial response that lasted 9.5 months in a patient with Erbb2 (Her2)-negative breast cancer cells with a FBXW7 mutation. In a preclinical study, the combination treatment of JQ1 and Ulixertinib (BVD-523) or PLX8394 resulted in greater decreased proliferation of HTLV-1 ATL cells with loss of function FBXW7 mutant S462P when compared to treatment with JQ1 alone [86]. Studies have shown that loss of FBXW7-mediated degradation of sterol regulatory element binding protein 1 (SREBP1) is associated with an increased expression of the IDH1 (Isocitrate Dehydrogenase (NADP(+)) 1) gene. Mutations in IDH1 can lead to downstream tumorigenic effects that alter histone and DNA methylation activities. Interestingly, the loss of FBXW7 and the presence of mutated IDH1 drastically increases radiation sensitivity of tumor cells. Therefore, co-occurrence of FBXW7 mutations (such as R505 or R465) along with mutated IDH1 may harbor a favorable prognosis [270].

## Conclusion

In summary FBXW7 is implicated in essential pro-survival signaling pathways and exhibits an inextricable link to cancer progression. In fact, FBXW7 is frequently inactivated or mutated in human cancers and inactivating mutations have been associated with resistance to therapies and poor prognosis for patients. Important challenges in studying FBXW7 remain due to the large number of cellular targets with different levels of binding affinity and the complex regulation of FBXW7 expression and its functions. For these reasons observations are often cell type specific and may not be generalized. Additionally, heterogeneity within tumors may further complicate interpretations of studies aimed to understand signaling pathways affected by FBXW7 inactivation. Another limitation is that reactivation of FBXW7 tumor suppressor functions, through gene editing or gene transfer therapy, is not easily feasible *in vivo.* However, the wide range of pro-oncogenic targets makes FBXW7 highly valuable for therapeutic targeting. Targeted therapies may offer new alternative treatments by taking advantage of FBXW7 mutations and specifically targeting tumor cells. Future studies will determine if targeting FBXW7 solely or using dual therapies that target FBXW7 and/or downstream or parallel pathways, is more beneficial to cancer treatment. The latter may prove more advantageous, as complete ablation of FBXW7 expression in combination with specific anti-cancer treatments, has been shown to desensitize cancer cells to death. This suggests that some FBXW7 activity may be required and that a reduction, but not complete loss, of FBXW7 activities may provide stronger anti-cancer therapy. By targeting FBXW7 indirectly, drug resistance may be relieved and allow for restoration of current drug therapies to work more efficiently. Future studies aimed to reactivate FBXW7 functions may take advantage of the fact that for the most part tumor cells are heterozygous and harbor both wild type and mutated copies. Since mutated FBXW7 has a dominant negative effect, it is possible to design or screen for small molecules that would selectively interact with mutated FBXW7 and disrupt dimerization. This approach may in theory sequester mutated FBXW7 freeing wild type FBXW7 protein to perform its tumor suppressor functions. Another possibility, and of special interest for future studies, is the search for the presence of neoantigens in mutated FBXW7, which may help develop novel cancer immunotherapies targeting cancer cells carrying specific mutations.

## Data Availability

All data generated or analyzed during this study are included in this published article. Molecular Cancer.

## Abbreviations

ATM: Ataxia-Telangiectasia Mutated
C/EBPδ: CCAAT/enhancer-binding protein-δ
CDK: Cyclin dependent kinase
ceRNA: Competing endogenous RNA
CRC: Colorectal carcinoma
CSN6: Signalosome subunit 6
DSB: DNA double strand breaks
DUB: Deubiquitinating enzymes
EMT: Epithelial mesenchymal transition
ERK: Extracellular signal-regulated kinase
GC: Gastric cancer
GSCs: Glioma stem-like cells
HCC: Hepatocellular carcinoma
HsSAS-6: Human spindle assembly abnormal 6
MEK: Mitogen-activated protein kinase
METTL: Methyltransferase-like
miRNAs: MicroRNAs
ncRNAs: Non-coding RNAs
NSCLC: Non-small cell lung cancer
PKC: Protein kinase C
PLK1: Polo like kinase-1
PRMT5: Protein arginine methyltransferase 5
Raf: Rapidly accelerated fibrosarcoma
Ras: Rat sarcoma virus
SCD1: Stearoyl coenzyme A desaturase
SREBP: Sterol Regulatory Element-Binding Protein
STAT3: Signal Transducer and Activator of Transcription 3
TNBC: Triple-negative breast cancer
WTAP: Wilms' Tumour 1-Associating Protein
XRCC4: X-Ray Repair Cross Complementing 4
